# Arsenic perception and signaling: The yet unexplored world

**DOI:** 10.3389/fpls.2022.993484

**Published:** 2022-09-02

**Authors:** Cristina Navarro, Micaela A. Navarro, Antonio Leyva

**Affiliations:** Department of Plant Molecular Genetics, Centro Nacional de Biotecnología-Consejo Superior de Investigaciones Científicas, Madrid, Spain

**Keywords:** arsenic signaling, abiotic stress, heavy metal contamination, phytoremediation, food safety, transporters, root growth

## Abstract

Arsenic is one of the most potent carcinogens in the biosphere, jeopardizing the health of millions of people due to its entrance into the human food chain through arsenic-contaminated waters and staple crops, particularly rice. Although the mechanisms of arsenic sensing are widely known in yeast and bacteria, scientific evidence concerning arsenic sensors or components of early arsenic signaling in plants is still in its infancy. However, in recent years, we have gained understanding of the mechanisms involved in arsenic uptake and detoxification in different plant species and started to get insights into arsenic perception and signaling, which allows us to glimpse the possibility to design effective strategies to prevent arsenic accumulation in edible crops or to increase plant arsenic extraction for phytoremediation purposes. In this context, it has been recently described a mechanism according to which arsenite, the reduced form of arsenic, regulates the arsenate/phosphate transporter, consistent with the idea that arsenite functions as a selective signal that coordinates arsenate uptake with detoxification mechanisms. Additionally, several transcriptional and post-translational regulators, miRNAs and phytohormones involved in arsenic signaling and tolerance have been identified. On the other hand, studies concerning the developmental programs triggered to adapt root architecture in order to cope with arsenic toxicity are just starting to be disclosed. In this review, we compile and analyze the latest advances toward understanding how plants perceive arsenic and coordinate its acquisition with detoxification mechanisms and root developmental programs.

## Introduction

Plants are constantly scouting their surrounding environment in the search for nutrients and water. However, soils contain multiple toxic metals and metalloids, some of them essential for plant growth in low amounts. Therefore, plants must display a battery of tightly regulated perception mechanisms that allow them to distinguish between beneficial or harmful elements, finely tuning their nutrient intake and metabolism or detoxifying through sequestering or extrusion. Roots are the first organs to sense soil composition and texture and consequently plants are constantly adapting root growth to the prevailing environment, reshaping its architecture to address a highly efficient nutrient capture mode but in parallel controlling the entry of toxic elements (Baligar et al., 1998; Lynch, 2021; Yadav et al., 2021). This is particularly delicate when nutrients and toxic compounds share the same transporter. Consequently, plants must be endowed with a precise control mechanism that senses toxic elements and activates a rapid and meticulous response to restrict or coordinate the entry of the toxic element to be metabolized, transported into the vacuole or extruded outside the cell. This response is extremely important in the case of arsenic detoxification. This metalloid is one of the most carcinogenic elements present in soils and waters used for human consumption or crop irrigation (IARC Working Group on the Evaluation of Carcinogenic Risks to Humans, 2012; Naujokas et al., 2013). Arsenic has been a persistent ancient stress for plants since the origin of life on Earth due to volcanic emissions, compromising plant growth and fitness (Zhu et al., 2014). This particularly affects rice, because it accumulates more arsenic in grain than any other grain crop (Zavala and Duxbury, 2008; Hojsak et al., 2015). This situation is extremely important in Asia where rice is the staple food of the majority of the population, and where people have been exposed to arsenic-contaminated waters for decades, what has been considered as the largest mass poisoning ever suffered by humanity in history (Sen and Biswas, 2013; Chakraborti et al., 2015). Furthermore, it has been predicted that the rise in temperatures due to climate change will double inorganic arsenic accumulation in rice grain, which will dangerously enhance the dietary exposure of millions of human beings to arsenic (Muehe et al., 2019). This extreme situation has raised interest in the design of new strategies to cope with arsenic contamination. Thus, understanding how plants sense and regulate the arsenic response not only would be crucial for phytoremediation strategies, but also to generate new plant varieties that show reduced arsenic accumulation in edible parts, sustaining crop production in contaminated soils.

The most abundant arsenic chemical species present in soils and groundwaters are arsenate [As(V)] and the reduced chemical form, arsenite [As(III)]. Among these two chemical species, the presence of As(III) in soils and subterranean waters is a global environmental problem. That is particularly serious in rice, being the most important gateway for arsenic to the human food chain (Zhao et al., 2010). This is due to the fact that rice is cultivated by flooding, which promotes the partitioning of arsenic from soil solids to pore-water, stimulating the breakup of As-bearing Fe(III) hydroxides leading to increased As(III) availability. As(III) is then rapidly incorporated into the vascular cells through symplastic intracellular transporters and rapidly accumulated in rice kernels. Recently, the study of the accumulation in plants of arsenic methylated forms produced by soil microorganisms (Lomax et al., 2012; Di et al., 2019), particularly the highly toxic dimethyl monothioarsenate (DMMTA), has raised research interest (Colina Blanco et al., 2021; Dai et al., 2021, 2022; Zhao et al., 2021; Pischke et al., 2022). However, the molecular mechanisms involved in uptake and translocation of these methylated arsenic species remain mostly unknown (Kerl et al., 2019).

Arsenic is extremely toxic to all living forms, causing the inactivation of multiple biological processes essential for life (Hughes, 2002; Finnegan and Chen, 2012; Shen et al., 2013) and therefore it can be predicted that all organisms must have evolved fast and efficient sensing mechanisms that coordinate the arsenic response. Essentially, both in prokaryotes and eukaryotes arsenic tolerance consists in a combination of arsenic uptake, extrusion or sequestration. In fact, the molecular components of arsenic uptake and detoxification in bacteria, yeast and plants have been characterized and extensively reviewed (Rosen, 1999, 2002; Tripathi et al., 2007; Zhao et al., 2010, 2021; Abbas et al., 2018; Yan et al., 2018; Garbinski et al., 2019; Bali and Sidhu, 2021; Mondal et al., 2022). Similarly, the mechanisms involved in arsenic detoxification in humans and other higher organisms are well known (Kumagai and Sumi, 2007). In bacteria, yeast and plants arsenic uptake and detoxification mechanisms are essentially similar. As(V), is structurally similar to phosphate (Pi) and therefore its uptake occurs through Pi-transporters these organisms (Rosenberg et al., 1977; Willsky and Malamy, 1980; Bun-ya et al., 1996; Yompakdee et al., 1996; Shin et al., 2004; Catarecha et al., 2007; Shen et al., 2012; Castrillo et al., 2013; Jiang et al., 2014). Once inside the cells, As(V) is quickly reduced to As(III) by the action of arsenate reductases (Mukhopadhyay and Rosen, 2002; Chao et al., 2014; Sánchez-Bermejo et al., 2014; Shi et al., 2016) and then rapidly extruded outside the cells by plasma membrane transporters (reviewed by Rosen, 2002; Garbinski et al., 2019; Zhao et al., 2021). In addition, As(III) can be sequestered by thiol-rich proteins, metallothioneins and phytochelatins, all of which display an extraordinary affinity for As(III). In yeast and plants, phytochelatins bind As(III) and these complexes are rapidly sequestered into the vacuole by the action of ABCC transporters (Ghosh et al., 1999; Ha et al., 1999; Schmöger et al., 2000; Mendoza-Cózatl et al., 2010; Song et al., 2010).

It is somewhat surprising that As(V) tolerance depends on its reduction to As(III), despite the fact that As(III) is more toxic than As(V). One possible explanation is that at the beginning of the history of Earth, As(III) was the prevalent chemical species in a reducing atmosphere which forced all living organisms to evolve strategies to cope with this chemical species. However, when the great oxygenation event occurred, As(V) became the most prevalent form in the biosphere (Oremland et al., 2009; Fru et al., 2019). In this context, it seems plausible that the detoxification of As(V) would take advantage of the preexisting As(III) detoxification system rather than generating a completely new detoxification system. In fact, a single enzymatic reaction catalyzed by an arsenate reductase converts As(V) into As(III) (Mukhopadhyay and Rosen, 2002; Rosen, 2002).

In addition to its reduction to As(III), it has been shown that some bacterial strains, isolated from highly arsenic-contaminated waters, exhibit increased preferential affinity for phosphate than for As(V) (Elias et al., 2012), suggesting that the affinity of the Pi/As(V) transporters could also be modulated as a strategy to cope with As(V). Changes in the Pi/As(V) affinity have been reported in the arsenic hyperaccumulator fern, *Pteris vittata*. In contrast to bacterial strains, in *Pteris*, the Pi/As(V) transporter shows higher affinity for As(V) than for Pi, leading to increased As(V) uptake and contributing to the extraordinary capacity of this fern to accumulate arsenic (Poynton et al., 2004; DiTusa et al., 2015).

Root architecture is an essential element in soil resource acquisition and, therefore, it is a primary determinant to prevent arsenic uptake. Arsenic exposure triggers specific developmental responses, being a major factor for plant survival to arsenic (Bahmani et al., 2016; Kumar et al., 2020; Yadav et al., 2021). Toward this, it is essential for plants to exhibit finely tuned regulatory circuits that integrate arsenic uptake, extrusion or sequestering with plant growth developmental programs, particularly in roots. However, sensing and signaling pathways that coordinate root growth plasticity with the presence of arsenic are mostly unknown. Here, we provide a comprehensive overview of the current understanding of arsenic signaling and the regulatory mechanisms in plants compared with those described in other organisms, in particular, bacteria and yeast.

## Arsenic perception

The identification of the primary arsenic sensor, if that exists, is one of the most important unresolved issues for the comprehension of the arsenic perception mechanisms in plants. Sensing is the first committed step in signaling pathways. Its understanding involves the identification of the sensor, as well as the signal perceived by the sensor. While the identification of the arsenic sensor in plants has remained elusive, we recently provide evidence toward the identification of the arsenic signaling molecule. Indeed, we have shown that As(III) is a key regulatory signal that regulates the arsenic response, even controlling the expression of the Pi/As(V) transporter in response to arsenic (Navarro et al., 2021). Notably, in line with the case in plants, in bacteria As(V) detoxification mechanisms are regulated by As(III), and likewise in yeast, As(III) is the arsenic signal as well (Xu et al., 1998; Di and Tamas, 2007; Kumar et al., 2015). However, As(III) and As(V) have similar geometries and therefore we cannot exclude the possibility that As(V) or other alternative mechanisms may also be involved in the activation of the arsenic response. Nonetheless, the fact that the first step in As(V) detoxification is reduction to As(III) by arsenate reductases, allowed bacteria, yeast and plants to conserve their arsenic detoxification mechanisms under As(III) regulation. Therefore, the existence in plants of a sensor specialized in As(III) perception is a seducing hypothesis that would allow plants to keep the arsenic response under control of As(III). In addition, As(III) provides higher selectivity than As(V) in arsenic signaling vs. Pi signaling, as As(V) displays high chemical similarity to Pi, and this is not the case with As(III).

In plants, several metal sensors have been described, mostly nutrient transporters, named “transceptors,” that sense their substrates (Ho et al., 2009; Dubeaux et al., 2018; Podar and Maathuis, 2022). Related to arsenic, the sulfur transporter SULTR1;2 has been proposed to act as a sulfur sensor conferring arsenic tolerance (Zheng et al., 2014; El-Zohri et al., 2015; Nishida et al., 2016), which is mediated by the metabolic activation of cysteine biosynthesis, resulting in increased thiol protein content, and consequently enhanced As(III) sequestration. Therefore, although we cannot exclude the possibility that As(III) transporters may also act as *bona fide* As(III)-transceptors, the potential nature of the arsenic sensor in plants can only be speculated based on the information obtained from other organisms, specially bacteria and yeast.

In bacteria, the sensor is the ArsR repressor protein that negatively regulates the *ars* operon involved in arsenic detoxification. ArsR is a small protein that contains a helix- turn-helix domain. It has been proposed that the binding of As(III) to ArsR triggers a conformational change in the DNA-binding site of the repressor that results in the dissociation from the *ars* promoters, allowing the transcription of the operon (Xu et al., 1998). Therefore, ArsR is considered a trans-acting repressor which senses As(III). Recently, a sophisticated modification of the ArsR sensor in the primitive bacteria *Paracoccus sp*. has been described (Chen et al., 2022). In this bacterial species the arsenic repressor AsR is translationally fused to the arsenate reductase ArsC. Therefore, the reduction of As(V) to As(III) occurs in the same protein that encodes ArsR and, as a result, the efficiency of ArsR binding to As(III) is significantly higher as it is more independent of diffusion barriers. Thus, the *ars* operon from this bacteria can be activated at lower As(III) concentrations, improving the sensitivity of the arsenic sensor to perceive the amount of arsenic inside the cell.

**Figure 1:**
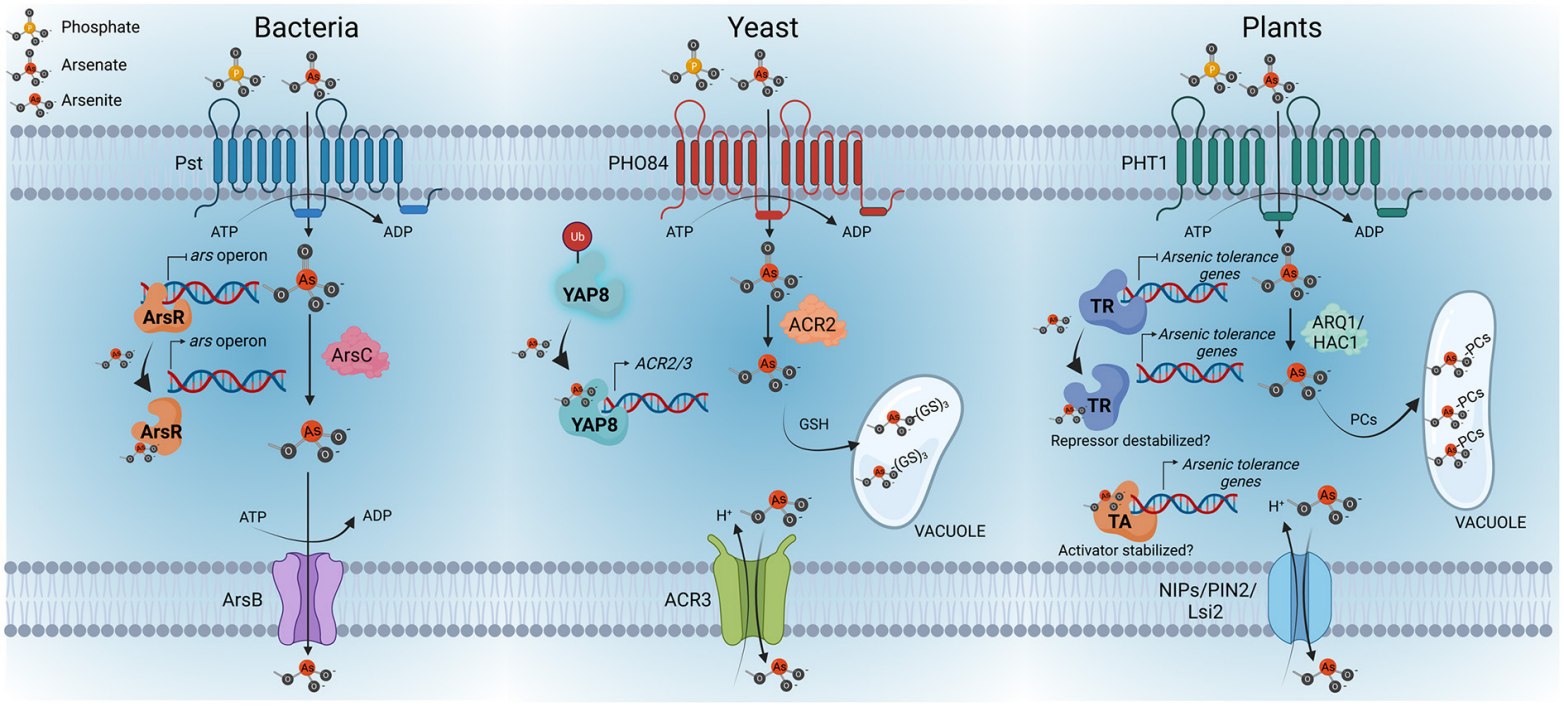
Overview of arsenic perception in bacteria, yeasts and plants. As(V) uptake is mediated by phosphate transport systems, Pst in bacteria, PHO84 in yeast and PHT1 in plants. Once inside the cells, As(V) is rapidly reduced to As(III); the latter is the key regulatory signal conserved among these kingdoms. The bacterial arsenic resistance (ars) operon is controlled by the repressor ArsR, which acts as the As(III) sensor protein. As(III) binding to ArsR triggers a conformational change that causes the repressor to dissociate from the promoter, enabling the transcription of the *ars* operon, activating the expression of ArsB—an As(III)-carrier protein—and ArsC—an arsenate reductase—. In yeast, Yap8 is the As(III) sensor protein. In the absence of arsenic, Yap8 is constantly being ubiquitinated (Ub) for proteasome-mediated degradation. However, Yap8-As(III) interaction results in the stabilization of Yap8, which leads to the activation of the expression of arsenic resistance genes, specifically, genes coding for the arsenate reductase ACR2 and the As(III)-extrusion pump ACR3. As(III) can also be conjugated with glutathione (GSH) and then sequestered into the vacuole of yeasts. Considering these findings in bacteria and yeast, it can be speculated that transcription factors could be the As(III)-sensor proteins in plants. The existence of a transcriptional repressor (TR) or activator (TA) of the arsenic response in plants and its stabilization by As(III) still needs to be explored. Similar to bacteria and yeast, the mechanisms of arsenic detoxification in plants involve As(V) reduction to As(III), mediated by arsenate reductases, specifically ARQ1/HAC1, and the consequent extrusion of As(III) through NIP/PIN2/Lsi transporters, as well as the conjugation of As(III) with phytochelatins (PCs) to form As(III)-cysteine-rich conjugates that can be transported and sequestered into vacuoles. Created with BioRender.com.

In yeast, the transcriptional activator Yap8 is a key regulatory protein of arsenic detoxification responses. Yap8 is a bZIP protein that in the absence of arsenic is constantly being degraded by the ubiquitin proteasome pathway (Di and Tamas, 2007; Kumar et al., 2015). In the presence of arsenic, Yap8 binds As(III), which results in the stabilization of the protein, leading to transcriptional up-regulation of the arsenic tolerance genes, specifically the genes coding for the arsenate reductase ACR2 and the As(III) extrusion pump ACR3 (Ilina et al., 2008). Interestingly, another member of this family of TFs is YAP1, which is considered the master regulator of the oxidative stress response and it also has a role in the detoxification of arsenic. However, there is no evidence of a direct interaction of YAP1 with As(III) (Menezes et al., 2008; Rodrigues-Pousada et al., 2010). In line with the findings described for yeast and bacteria, transcription factors emerge as candidates for As(III) sensors in plants (Figure 1). Further research work must be done to identify the *bona fide* arsenic sensor or some other molecular mechanisms involved in arsenic sensing in plants.

## Arsenic signaling mechanisms

Plant response to arsenic requires a tight coordination of several strategies implying a complex regulatory network inextricably intertwined with regulatory pathways involved in coping with other abiotic stresses. This considerable complexity is further increased due to the interconnection between different toxic elements and nutrients. For instance, selenium (Se) competes with sulfur (S), As(V) with Pi, or As(III) with silicon (Si) (Korshunova et al., 1999; Thomine et al., 2000; Shin et al., 2004; Sors et al., 2005; Ma et al., 2008; Zhao et al., 2010; Himeno et al., 2019). Furthermore, bidirectional transporters of several micronutrients like boron (B) and trace elements like Si, antimony (Sb) and Se (Pommerrenig et al., 2015) are also involved in arsenite extrusion. Therefore, plants have evolved sophisticated and promiscuous mechanisms to deal with toxic compounds in the presence of nutrients, hindering the identification of the specific signal transduction pathways participating in the arsenic response. In any case, there have been several reports describing transcriptional and post-transcriptional regulators in arsenic signaling. Nonetheless, master regulatory components of the transcriptional response to arsenic have not been unearthed.

### Transcriptional regulation

The first transcription factor involved in arsenic signaling has been WRKY6 (Castrillo et al., 2013). Due to its high chemical similarity with Pi, As(V) uses the Pi transporters to enter into the plant cell. To cope with this, once plants perceive As(V), the transcription of the Pi-transporter *PHT1;1* is rapidly repressed, to impede the entry of the metalloid and Pi inside the cell (Castrillo et al., 2013). Similarly to As(V), high Pi also represses the Pi transporter, which is also mediated by WRKY6, suggesting that there are some overlapping signaling components of the As(V) and Pi response. However, WRKY6 is regulated by degradation in response to low Pi (Chen et al., 2009; Ye et al., 2018) while it is transcriptionally regulated by As(III) in response to arsenic, providing an As(V)/Pi-independent arsenic regulation of the Pi transporter. The activation of *WRKY6* by As(III) and its degradation by low Pi provides a fine-tuned regulatory loop to modulate As(V) uptake, maintaining the regulatory machinery ready to activate the expression of the Pi-transporter as soon as As(V) disappears from the medium.

As(III) binds thiol groups of proteins, inhibiting their enzymatic activities, which provokes the generation of reactive oxygen species (Nahar et al., 2022). Oxidative stress damages various cellular structures, leading to a general unfolded protein response. In this context, glutathione plays a central role in protecting plants from oxidative stress, providing an adequate redox environment for enzyme activity and membrane stability (García-Giménez et al., 2013; Diaz-Vivancos et al., 2015; Dorion et al., 2021). In addition, glutathione itself, or as a precursor of phytochelatins, is also essential for As(III) sequestering into the vacuole and therefore plants exposed to arsenic are particularly sensitive to oxidative stress (Ha et al., 1999; Schmöger et al., 2000). Consequently, transcription factors such as SLIM1, a master regulator involved in sulfur uptake (Maruyama-Nakashita et al., 2006), are critical for arsenic tolerance, since sulfur is a constituent of cysteine and therefore of glutathione (Jobe et al., 2021). Similarly, other transcription factors involved in arsenic tolerance are also essential for plants to handle other stresses such as drought or temperature, both associated with oxidative stress. Accordingly, drought stress, which increases abscisic acid (ABA) concentration, controls arsenic and cadmium (Cd) accumulation (Fan et al., 2014; Abdel-Haliem et al., 2017; Saha et al., 2021). Interestingly, both toxic elements are sequestered into the vacuole complexed with glutathione and phytochelatins, suggesting a clear overlap of some regulatory elements of the Cd and As(III) response. The prevention of Cd accumulation in response to ABA is known to be mediated by the interaction of the ABA-signaling transcription factor ABI5 with MYB49, another transcription factor that upregulates the expression of transcriptional activators of the *IRT1* transporter involved in Cd uptake (Zhang et al., 2019). The formation of the heterodimer ABI5-MYB49 inhibits the binding of MYB49 to the promoter region of its target genes, leading to the repression of the Cd transporter. However, in the context of As(III) uptake and signaling, ABA seems to operate through a different mechanism. In rice, ABA negatively regulates the expression of *OsARM1*, a repressor of *Lsi1* and *Lsi2* transporters, responsible for As(III) uptake through the exodermis and endodermis (Wang et al., 2017; Hu et al., 2020). Consequently, knockout of *OsARM1* enhances arsenic accumulation in the above ground tissue. Furthermore, some studies hint that ABA upregulates the expression of the Pi-transporter repressor *WRKY6*, which in turn may lead to the suppression of As(V) uptake (Huang et al., 2016; Song et al., 2016). The mechanism involved in *OsARM1* transcriptional repression mediated by ABA remains largely unclear.

A recent study has identified an arsenic-inducible myb transcription factor, MYB40 as an important regulator conferring arsenic resistance in Arabidopsis (Chen et al., 2021). *myb40* mutant alleles showed a constitutive activation of the Pi/As(V) transporter *PHT1;1*, similar to *wrky6* mutants (Castrillo et al., 2013). In addition, the transcriptional upregulation in response to arsenic of phytochelatin synthases and ABCC transporters involved in As(III) transport into the vacuole was significantly increased in the *myb40* mutants (Chen et al., 2021). Further experiments will be required to determine whether or not MYB40 is a key regulatory factor involved in arsenic sensing and signaling of the arsenic detoxification mechanisms.

Also recently, genome wide approaches have proven useful to identify key regulatory proteins involved in arsenic signaling. For instance, using a library of artificial microRNAs (amiRNAs) targeting diverse genes, several CBF transcription factors have been identified as negative regulators of arsenic tolerance, probably through direct regulation of Pi transporters, (Xie et al., 2021). Likewise, transcriptomic analyses of two Arabidopsis accessions with differential response to arsenic treatment provided several candidate genes, including transcription factors from the B-box (BBX), NAC or MYB families (Khare et al., 2022). The candidate genes identified in these studies should be further characterized in order to assess their putative role in the regulation of the response to arsenic. Nevertheless, genome-wide association studies using an Arabidopsis collection of accessions proved to be essential tools for the identification of key regulatory factors involved in the arsenic response. Therefore, arsenic tolerance is controlled by a collection of key regulatory proteins that contribute to achieve the most efficient arsenic tolerance phenotype. However, we must consider the possibility that a master regulator of the arsenic response may exist as it has been previously described in yeast and bacteria (Xu et al., 1998; Kumar et al., 2015). Further analysis will determine whether or not some of these regulatory proteins act as master regulators, coordinating the responses that contribute to attain the final arsenic tolerance.

### Post-transcriptional regulation

The control of protein homeostasis and post-translational modifications (PTM) are also essential components for stress survival across different species (Zhang et al., 2015; Kosová et al., 2021). Strikingly, there is growing evidence that state changes of key regulatory proteins are associated with more dramatic functional alterations for the cell than protein abundance (Needham et al., 2019; Ochoa et al., 2019; Mehnert et al., 2020; Grossbach et al., 2022). Actually, several specific phosphorylation states have been correlated to a number of stress resistance traits in the context of a novel high-quality multi-omics QTL study in yeast, showing, for the first time at a global scale, the central importance of protein phosphorylation to adapt stress responses in living organisms (Grossbach et al., 2022). However, the study of protein state changes in response to arsenic, including PTMs, is poorly developed, although there are some examples pointing to their relevance in arsenic signaling. Thus, it has been shown that MAPKs are activated in response to As(III), suggesting their implication in arsenic signaling (Rao et al., 2011). Additionally, recent reports indicate that PTMs modulate arsenic transport (Tang and Zhao, 2021). The activity of PHO1, a transporter involved in As(V) xylem loading, and INT2/4, inositol transporters also permeable to As(III) for phloem loading may be modulated by phosphorylation. Moreover, PHT1 and PHO1 transporters also undergo several regulatory PTMs during their intracellular trafficking to the plasma membrane and Golgi/trans-Golgi network, respectively, including ubiquitination and phosphorylation (reviewed by Pan et al., 2019). For instance, the C-terminus phosphorylation of the PHT1;1 and PHT1;4 transporters, involved in Pi/As(V) transport, by casein kinase CK2 keeps them anchored to the ER under Pi-sufficient conditions (Bayle et al., 2011; Chen et al., 2015). This additional level of control for the activity of PHT1 proteins could also be used to regulate PHT1-mediated As(V) uptake. Similarly, it has been shown that the activity of the As(III) transporter NIP1;1 can be positively regulated through phosphorylation by the Ca-dependent protein kinase by CPK31 (Ji et al., 2017), pointing to the importance of PTMs to modulate As(III) arsenic uptake. As we mentioned above, the transcriptional repressor *OsARM1*, involved in the control of the As(III) transporters *Lsi1* and *Lsi2*, is regulated by ABA. Similarly, Cd uptake is also regulated by ABA which is mediated by phosphorylation-dephosphorylation of the ABA-responsive transcription factor ABI5 (Fujii et al., 2009; Zhang et al., 2019). A similar strategy could also be operating to control *OsARM1* transcriptional expression, limiting arsenic accumulation in plants. However, changes in the phosphorylation status of OsARM1 are currently unknown and the role of ABI5 in *OsARM1* expression requires further investigation.

Ubiquitin and SUMO conjugations are also major post-translational modifiers controlling protein homeostasis in response to stress (Vierstra, 2012). However, there are still no reports regarding the implication of sumoylation in the arsenic response. Interestingly, two studies uncovered the participation of F-boxes in arsenic responses suggesting that ubiquitination is involved in arsenic signaling. One study comprised a GWA analysis of *Arabidopsis* ecotypes, leading to the identification of ASRF, a F-box protein involved in maintaining phosphate homeostasis under As(V) stress (Peña-Garcia et al., 2021). In this case, the *asrf* mutant showed an arsenic-sensitive phenotype linked to a higher activation of Pi/As(V) transporters compared to the wildtype, highlighting the importance of F-box proteins-mediated ubiquitination for dealing with arsenic toxicity. Therefore, it can be hypothesized that in response to arsenic, ASRF mediates the degradation of transcription factors involved in the activation of Pi/As(V) transporters, leading to the suppression of As(V) uptake. In addition, a second study showed that another F-box, named PHIF1, targets PHR1, the key activator of Pi/As(V) transporters, for ubiquitination and subsequent degradation in response to arsenic (Navarro et al., 2021).

Small non-coding RNAs, including microRNAs (miRNAs) have an emerging role as regulatory components, post-transcriptionally modulating gene activity to cope with heavy metal stress (Ding et al., 2020). In this sense, the innovation in next-generation sequencing platforms has allowed the identification of a number of heavy metal-regulated miRNAs as essential regulatory components for plant tolerance to As, Cd, mercury (Hg), aluminum (Al) and lead (Pb) (Chen et al., 2011; Liu and Zhang, 2012; Han et al., 2016; Shen et al., 2017; Ding et al., 2018; Wu et al., 2018; Gao et al., 2019). In the context of arsenic, microarray profilings of miRNAs performed in *B. juncea*, and in natural rice accessions exposed to As(V) or As(III) species, led to the identification of tens of differentially-expressed miRNA (Srivastava et al., 2012; Sharma et al., 2015). Among them, it is worth mentioning miR399 and miR528. miR399 is known to be induced in Pi-starvation conditions, acting as a repressor of PHO2 (an E2 ubiquitin conjugase), which modulates the degradation of the Pi/As(V) transporters PHO1 and PHT1 (Aung et al., 2006; Bari et al., 2006; Liu and Zhang, 2012). Interestingly, in the same study they found that miR528 overexpressing lines exhibit As(III)-sensitivity as a consequence of an overall impairment of As(III) uptake, translocation and tolerance mechanisms probably through targeting *Lsi2* As(III) transporter (Liu et al., 2015). Thereby, most of the arsenic stress-responsive miRNAs described so far have not been fully characterized for their molecular function in arsenic tolerance.

## Hormones, root architecture, and arsenic response

The presence of arsenic exerts a profound effect on root architecture that should be tightly coordinated with the detoxification machinery. It is known that arsenic reshapes the spatial configuration of roots mostly due to repression of root hair elongation and root growth arrest, particularly of the main root, altering the boundaries of meristematic and elongation zones (Catarecha et al., 2007; Fattorini et al., 2017; Tu et al., 2021). Indeed, root apical meristem is the major absorption area involved in As(III) uptake (Ashraf et al., 2019). It is somehow surprising that, in contrast to our results (Catarecha et al., 2007), in two independent studies, it was found that arsenic induces root hair elongation (Bahmani et al., 2016; Kumar et al., 2020). In both studies the experimental conditions were different from those used in Catarecha et al. (2007) and it could be possible that in these conditions arsenic availability was lower, allowing the plant to detoxify the arsenic, increasing its accumulation and consequently releasing the suppression of root hair elongation. In line with this, some arsenic-tolerant accessions of Arabidopsis and *Holcus lanatus* reduced As(V) uptake due to a suppression of the high-affinity Pi uptake system, but displayed increased arsenic accumulation (Meharg and Macnair, 1990, 1991, 1992; Bleeker et al., 2002; Meharg and Hartley-Whitaker, 2002; Catarecha et al., 2007; Castrillo et al., 2013). These observations indicate that arsenic detoxification mechanisms and root growth responses are intimately intertwined.

Hormone distribution along roots could be a critical factor in the integration of arsenic perception with root architecture. Different hormones, like jasmonic acid, salicylic acid, brassinosteroids, ethylene, gibberellins, and strigolactones have been involved in arsenic tolerance, although their effect is mostly due to their capability to control oxidative stress (Surgun-Acar and Zemheri-Navruz, 2019; Coelho et al., 2020; Kaya et al., 2020; Mostofa et al., 2021a,b; Samanta et al., 2021; Singh et al., 2021). However, several experiments point out that ABA, auxin and cytokinins have a specific role in root growth adaptation to arsenic toxicity besides oxidative stress mitigation.

ABA modulates root developmental programs independently of stress conditions through the control of long-distance transport of the hormone (Zhang et al., 2021). Arsenic treatment increases ABA accumulation by unknown mechanisms and consequently upregulates genes involved in ABA biosynthesis and signaling (Huang et al., 2012; Yu et al., 2012; Hu et al., 2020). Interestingly, upregulation of ABA-responsive genes in response to arsenic only occurs in ecotypes that show an arsenic-tolerant phenotype but not in those exhibiting arsenic sensitivity (Fu et al., 2014), strongly supporting that ABA mediates arsenic tolerance. Accordingly, ABA also increases hyperaccumulation of glutathione and phytochelatins (Stroiński et al., 2010; Song et al., 2016; Shi et al., 2019). Furthermore, as we mentioned above, ABA negatively regulates the expression of *OsARM1*, a repressor of *Lsi* transporters involved in As(III) transport throughout the endodermis, increasing arsenic accumulation in the above ground tissue, further supporting its role in arsenic tolerance (Wang et al., 2017). Another facet of ABA action is that this phytohormone has been recently found to be involved in the formation of the endodermis barriers and therefore may alter arsenic translocation into the aerial part (Brookbank et al., 2021). In summary, all these observations support that ABA could be involved in the control of root architecture in response to arsenic, being a major factor in the integration of root development with arsenic uptake and translocation.

Cytokinins, which are intimately linked to root growth inhibition, are involved in the repression of the Pi/As(V) transporters, probably coordinating As(V) uptake with root elongation, being a major factor in the coordination of arsenic uptake and detoxification with uptake (Mohan et al., 2016). Indeed, cytokinins content is associated with increased arsenic content in the arsenic hyperaccumulator *Pteris cretica var. nervosa*, so that an increase in bioactive cytokinins and a decrease of inactive forms of these hormone correlates with the accumulation of arsenic (Zhang et al., 2017). However, this correlation was not found in the non-hyperaccumulator *Pteris ensiformis*. Therefore, it appears that the cytokinin-arsenic interplay has been rewired during evolution and supports that these hormones are important for tolerating high intracellular arsenic content, having a significant impact on plant growth.

Auxin is also essential for root growth plasticity in response to arsenic (Tu et al., 2021). Furthermore, the polar auxin transporter PIN2 has been involved in As(III) extrusion (Ashraf et al., 2019), suggesting that the presence of arsenic may somehow modulate auxin distribution and therefore root architecture. Accordingly, arsenic exposure reduces auxin levels in adventitious and lateral roots in Arabidopsis and rice, affecting biosynthesis and transport of this hormone, ultimately giving rise to a modified root architecture (Krishnamurthy and Rathinasabapathi, 2013; Fattorini et al., 2017; Tripathi et al., 2021). Interestingly, the amount of auxin in roots is altered in sulfur-starvation conditions where phytochelatin biosynthesis is arrested suggesting that sulfur may act as a key regulator that integrates root architecture with arsenic detoxification due to changes in auxin gradients.

In this context, an exciting field of interest is the contribution of arsenic-resistant plant growth-promoting rhizobacteria (PGPR) and other rhizospheric microorganisms to attenuate arsenic phytotoxicity and promote root development. Indeed, these microorganisms promote nutrient (Pi, N, Fe) uptake, and modulate arsenic bioavailability and phytohormone levels. Indeed, they increase auxin levels by the production of indole-3-acetic acid (IAA), and reduce ethylene content by the production of 1-aminocyclopropane-1-carboxylate (ACC) deaminase, thus resulting in increased root growth (reviewed by Upadhyay et al., 2018; Mondal et al., 2021; Kumar et al., 2022). Further experiments will be required in order to determine whether these bacteria are able to activate the arsenic signal transduction pathway.

**Figure 2:**
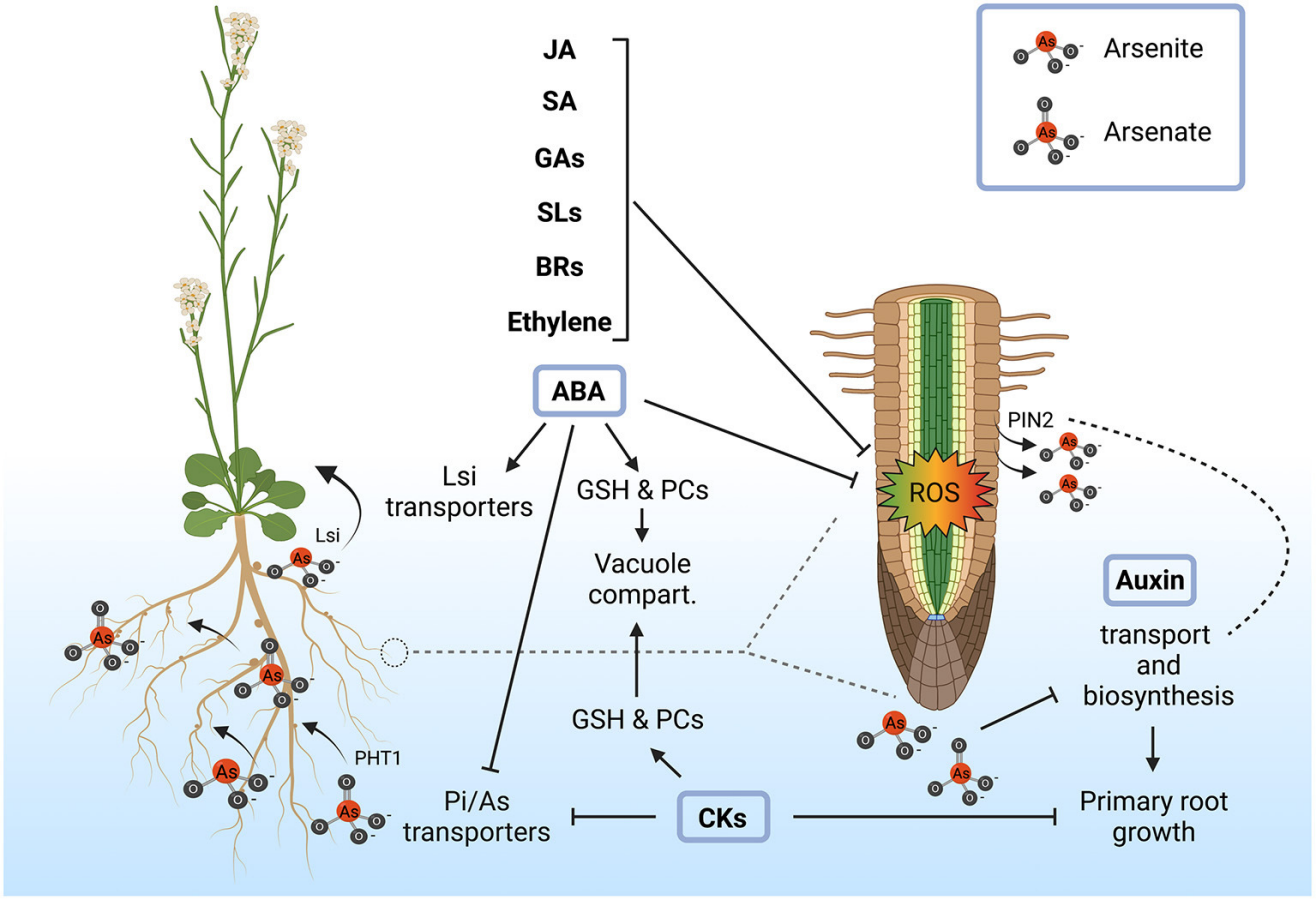
Role of plant hormones in the alleviation of arsenic toxicity and root plasticity. Exogenous application of abscisic acid (ABA), ethylene, brassinosteroids (BRs), strigolactones (SLs), gibberellins (GAs), salicylic acid (SA) and jasmonic acid (JA) enhances plant resistance to arsenic stress through the mitigation of reactive oxygen species (ROS). ABA, cytokinins (CKs) and auxins are more directly implicated in arsenic detoxification and tolerance mechanisms by the regulation of arsenic transport and accumulation together with the modulation of root architecture. ABA increases arsenic tolerance mainly by increasing glutathione (GSH) and phytochelatins (PCs), which in turn promotes the accumulation of arsenic in the vacuole. In rice, ABA may also be involved in the activation of Lsi transporters, leading to As(III) translocation into the aerial part. Similarly, CKs also contribute to increase GSH and PCs synthesis, enhancing vacuolar sequestration of arsenic. In addition to this, it has been shown that CKs contribute to the repression of Pi/As(V) transporters (PHT1) in response to arsenic in order to limit As(V) uptake and this mechanism is coordinated with the suppression of primary root growth. On the other hand, arsenic most probably interferes with auxin transport and biosynthesis, due to the fact that As(III) is extruded by the auxin transporters PIN2, reshaping root architecture. Created with BioRender.com.

In summary, hormones, particularly ABA, cytokinins and auxin, play an essential functional role in the adaptation of root architecture to arsenic uptake rate and detoxification capacity (Figure 2). Further experimental work will be required to determine the underlying molecular mechanisms in order to understand how plants integrate the presence of arsenic with root growth developmental programs mediated by hormones.

## Conclusion

Arsenic contamination in edible crops urgently needs a profound understanding of the molecular mechanisms underlying arsenic perception and signaling. However, arsenic response comprises a complex regulatory network intertwined with other stresses and developmental programs that makes the identification of key regulatory factors and sensor proteins an arduous task.

The identification of As(III) as a key signal that controls transcriptional regulation of the arsenic response prepares the ground for the identification of sensors and regulatory factors involved in arsenic perception and signaling. In addition, an important aspect of arsenic tolerance response that must be approached is the characterization of root growth adaptation to the presence of the metalloid. Roots are the first organs to sense arsenic, which may be present in different concentration gradients, leading to the activation of local and systemic arsenic responses in the root that must be characterized. Therefore, it is an urgent matter to study how roots transduce arsenic perception into root morphological responses integrated with tolerance mechanisms. In addition, further characterization of the root microbiota that modulates arsenic bioavailability and root growth adaptation to the presence of arsenic will be crucial to develop arsenic resistant crops and to aid phytoremediation strategies. Moreover, due to climate change prediction, it is also imperative to characterize the effect of light intensity, temperature, drought and salt, as essential factors that will have a dramatic impact on plant arsenic accumulation. All these actions will provide an integral overview of plant arsenic tolerance.

In conclusion, the characterization of the mechanisms involved in arsenic sensing and signaling is currently in a very preliminary stage and further in-depth studies are needed. However, the new genome-wide techniques for transcriptome and proteome profiling and gene editing techniques allow the exploitation of natural variation at an unprecedented scale, thereby predicting a thriving future in the comprehension of the molecular basis underpinning arsenic response in plants.

## Author contributions

AL conceived and wrote the manuscript with contributions for writing and reviewing from MAN and CN. All authors contributed to the article and approved the submitted version.

## Funding

This work was supported by fellowships from the Spanish Ministry of Science and Innovation (MICINN) and Severo Ochoa Centers of Excellence Grant Programme to CN, and by fellowships from Fulbright and FPU Ph.D. fellowship to MAN. This work was funded by Grants BIO2014-55741-R and BIO2017-87524-R, PID2021-125289OB-100 funded by MCIN/AEI/10.13039/501100011033, and Grant 2020AEP013 funded by the Spanish National Research Council (CSIC).

## Conflict of interest

The authors declare that the research was conducted in the absence of any commercial or financial relationships that could be construed as a potential conflict of interest.

## Publisher's note

All claims expressed in this article are solely those of the authors and do not necessarily represent those of their affiliated organizations, or those of the publisher, the editors and the reviewers. Any product that may be evaluated in this article, or claim that may be made by its manufacturer, is not guaranteed or endorsed by the publisher.
